# When stress and development go hand in hand: main hormonal controls of adventitious rooting in cuttings

**DOI:** 10.3389/fpls.2013.00133

**Published:** 2013-05-14

**Authors:** Cibele T. da Costa, Márcia R. de Almeida, Carolina M. Ruedell, Joseli Schwambach, Felipe S. Maraschin, Arthur G. Fett-Neto

**Affiliations:** ^1^Plant Physiology Laboratory, Center for Biotechnology and Department of Botany, Federal University of Rio Grande do Sul, Porto Alegre, Rio Grande do Sul, Brazil; ^2^Institute of Biotechnology, University of Caxias do Sul, Caxias do Sul, Rio Grande do Sul, Brazil

**Keywords:** adventitious rooting, auxin, receptors, jasmonic acid, cytokinin, nutrition, microRNAs, hormonal crosstalk

## Abstract

Adventitious rooting (AR) is a multifactorial response leading to new roots at the base of stem cuttings, and the establishment of a complete and autonomous plant. AR has two main phases: (a) induction, with a requirement for higher auxin concentration; (b) formation, inhibited by high auxin and in which anatomical changes take place. The first stages of this process in severed organs necessarily include wounding and water stress responses which may trigger hormonal changes that contribute to reprogram target cells that are competent to respond to rooting stimuli. At severance, the roles of jasmonate and abscisic acid are critical for wound response and perhaps sink strength establishment, although their negative roles on the cell cycle may inhibit root induction. Strigolactones may also inhibit AR. A reduced concentration of cytokinins in cuttings results from the separation of the root system, whose tips are a relevant source of these root induction inhibitors. The combined increased accumulation of basipetally transported auxins from the shoot apex at the cutting base is often sufficient for AR in easy-to-root species. The role of peroxidases and phenolic compounds in auxin catabolism may be critical at these early stages right after wounding. The events leading to AR strongly depend on mother plant nutritional status, both in terms of minerals and carbohydrates, as well as on sink establishment at cutting bases. Auxins play a central role in AR. Auxin transporters control auxin canalization to target cells. There, auxins act primarily through selective proteolysis and cell wall loosening, via their receptor proteins TIR1 (transport inhibitor response 1) and ABP1 (Auxin-Binding Protein 1). A complex microRNA circuitry is involved in the control of auxin response factors essential for gene expression in AR. After root establishment, new hormonal controls take place, with auxins being required at lower concentrations for root meristem maintenance and cytokinins needed for root tissue differentiation.

## INTRODUCTION

If flowering is a key developmental process for sexual reproduction in plants, adventitious rooting (AR) occupies a central role in asexual propagation. Forestry, horticulture, and fruit crops depend to a large extent on the successful establishment of roots in cuttings and other propagules. Clonal propagation is of particular relevance to forestry, since genetic improvement in long lived species with large generation cycles is often limiting. Genetic gains from interspecific hybridization, mutations, and transgenic events can be captured and multiplied faster and more efficiently based on clonal propagation through AR of cuttings. Overall, the main application of AR is propagation by cuttings and its derived techniques adapted to clonal garden greenhouses and *in vitro* cultures, minicuttings and microcuttings, respectively (Assis et al., 2004). Therefore, rather than looking into the examples of developmentally programmed AR in intact plants, the focus of the present review is on AR of severed organs or in response to stressful conditions, such as flooding.

Most research on AR has been centered on the role of phytohormones, mainly auxins, and cutting physiological conditions. The role of stress responses associated with cutting severance and the relevance of mother plant status has often received less attention, although a shift in focus has been clearly taking place in the last two decades or so. Wound responses associated with cutting severance are integrated, and often necessary, in the steps leading to AR, and mother plant status is a key determinant of rooting propensity of cuttings derived from it. Therefore, the control of environmental variables of stock plants is rather relevant for the clonal propagation process. Clearly, a fundamental aspect governing AR responses to external and internal stimuli is cellular competence to respond. This developmental capacity to respond is responsible for many of the failures to obtain AR in mature cuttings, even upon careful manipulation of environmental variables and phytohormones that can modulate rooting.

The concept of adventitious root is based essentially on anatomical origin. Adventitious roots are formed in stems, leaves and non-pericycle tissue in older roots, differing from primary roots, of embryonic origin, and lateral roots, which are derived from the pericycle layer (Li et al., 2009a). There are two main patterns of adventitious root development: direct and indirect. The tissues involved in the process of root development are most frequently the cambium and vascular tissues, which undergo the first mitotic divisions, leading directly to root primordia in the first pattern. In the indirect pattern of AR, albeit the same tissues often take part, the formation of a callus is observed prior to differentiation of root primordia. In both cases, before root primordia become distinguishable, clusters of usually isodiametric cells are formed (meristemoids; Altamura, 1996). In the indirect pattern of AR, a bottleneck is frequently observed, i.e., the establishment of an effective vascular connection between the newly formed root primordia and the stem. Poorly connected vasculature with the stem leads to non-functional roots, with negative consequences for cutting survival (Fleck et al., 2009).

Adventitious rooting is a complex process that can be affected by numerous variables, both internal and external. A large body of evidence has supported the existence of successive physiological phases in the process of adventitious root development, each with specific requirements that can even be antagonistic, but operate in complementary fashion. The most widely recognized AR phases are induction, initiation, and expression (Kevers et al., 1997).

The induction phase in cuttings or detached organs, such as leaves, is generally marked by the immediate consequences of the wounding response caused by severance. It encompasses the first hours after cutting removal, with a local increase in jasmonate, phenolic compounds and auxin at the cutting base, often associated with a transiently lower peroxidase (EC 1.11.1.7) activity, and the establishment of a sink for carbohydrates in the same zone (Schwambach et al., 2008; Ahkami et al., 2009). Peroxidases are heme-containing enzymes with catalytic action on diverse organic compounds, including indole-3-acetic acid (IAA), and their activity has been used as a biochemical marker of the rooting phases (Corrêa et al., 2012a). The induction phase is devoid of visible cell divisions and involves reprograming of target cells to the following establishment of meristemoids, which takes place in the initiation phase. Studying AR in apple, De Klerk et al. (1999) launched the concept of an early phase of dedifferentiation (0–24 h), taking place before the induction phase. In the concept of the present review, the dedifferentiation phase postulated by De Klerk et al. (1999) corresponds to the early steps of the induction phase. During initiation, besides cell divisions, meristemoids and development of root primordia, often a lower auxin and phenolic concentration and higher peroxidase activity are observed. The expression phase corresponds to the growth of root primordia through the stem tissues and the establishment of vascular connections between the newly formed root and the original stem cutting. For simplification purposes, it is not uncommon to join the initiation and expression phases under a single denomination of formation phase (Fett-Neto et al., 1992).

These overall changes in phytohormone balance, along with other less predominant but not unimportant changes to be discussed ahead, trigger a sequence of gene expression events that leads to proteomic changes, culminating with new root differentiation. Considering different systems and the fragmentary information available, these molecular events of gene expression and gene product accumulation can be putatively summarized in chronological sequence as follows: wounding and water balance stress-related, carbohydrate sink establishment, auxin transport systems, cell wall degradation and assembly, transcription factors involved in cell fate determination, replication machinery, transcription factors with roles in growth and differentiation (Brinker et al., 2004; Sorin et al., 2005; Ahkami et al., 2009).

At the molecular level, including the participation of various phytohormones, considerably more knowledge is available on lateral root development. Certainly, there is at least some overlap between the processes of lateral root development and AR. Most of the similarities include the requirement for an initial auxin increase, followed by a reduction, the participation of auxin transporters, cell wall dynamics, and the activity of specific transcription factors in these processes. Root growth responses to nutrient gradients, such as nitrate and phosphate (Desnos, 2008), seem to be another feature apparently shared between lateral and adventitious roots (Schwambach et al., 2005). Lateral root development depends at least partially on auxin activation of founder cells in the pericycle at the primary root differentiation zone, possibly mediated by an interaction of auxin with its receptor TIR1 (transport inhibitor response 1; Petricka et al., 2012). The role of root development inhibitors, such as cytokinins and strigolactones, which will be discussed ahead, also seems to be shared between lateral root development and AR. However, besides the usual histological origin, other very important differences exist between lateral and adventitious root development, most likely related to the often associated wound response and particular reorganization of auxin transport systems in the latter.

Although AR in intact plants may take place in certain conditions, such as flooding or programmed development, the typical AR features of stress signaling and major shifts in root–shoot correlative influences are usually present in excised plant parts, such as cuttings, hypocotyls and leaves. Rooting protocols based on pre-etiolated intact seedlings, commonly used to investigate AR in *Arabidopsis thaliana*, have roots formed mostly from the pericycle, which extends from primary roots into the hypocotyls of young seedlings, and do not face stresses capable of disrupting root–shoot correlative influences. A comparison of an intact seedling system with de-rooted older plants or with rooting of petioles of detached leaves showed significant differences, not only in root founding tissues, but also in auxin requirements, sensitivity, and rooting mutant phenotypes (Corrêa et al., 2012b). The fact that lateral and adventitious root developments have fundamental functional differences can be further highlighted by the opposite effects of ethylene on both processes, observed in studies with tomato (Negi et al., 2010). Perhaps the pathways leading to adventitious versus lateral root development could be viewed as different roads, which may intertwine in some portions, and end up leading to the same destination, i.e., new roots.

## MOTHER PLANT STATUS – DEVELOPMENTAL COMPETENCE TO RESPOND AND THE RIGHT SUPPLIES FOR THE HURDLES AHEAD

In vegetative propagation, a mother plant provides cuttings with improved selected characteristics, and the formation of new adventitious roots is essential for the restoration of the whole plant condition. Physiological and biochemical quality of mother plants, in addition to their genetic makeup, could limit rooting performance of cuttings derived therefrom (Osterc, 2009). The physiological condition of mother plants is directly affected by the environment in which they were raised or to which they were exposed, including light and temperature conditions, water and nutrient supplies (Moe and Andersen, 1988). Endogenous auxin, carbohydrate content, mineral nutrients, and other biochemical components, such as phenolics that could act as rooting co-factors or auxin transport modulators, may be affected by environmental factors and are transferred from the stock plants to the propagules when the cutting is severed. The content, metabolism, and interactions of these metabolites and components will influence early responses to wound and root induction of cuttings.

Auxin endogenous concentration varies over the course of rooting phases, and is needed at higher concentration during the induction phase for proper rooting (Kevers et al., 1997). In this context, high auxin content immediately after cutting severance originating from the mother plant, may result in improved rooting. As far as light treatments are concerned, shade conditions (low red:far-red ratios) induced auxin biosynthesis and increased IAA levels in *Arabidopsis* seedlings (Tao et al., 2008). Light availability and quality have been shown to affect auxin transport rate and its predominant anatomical path in the stem (Morelli and Ruberti, 2002). Sorin et al. (2005) described an interaction between light and auxin metabolism affecting *Arabidopsis* rooting. Mutants with low rooting capacity (*ago1*) had upregulated light responses and disturbed auxin homeostasis.

Mineral nutrition of stock plants is an important factor in determining AR capacity. The biosynthesis of one of the main auxin precursors, the amino acid tryptophan, requires zinc (Blazich, 1988; Marschner, 1995), which is also a structural component of the auxin receptor ABP1 (Auxin-Binding Protein 1; Tromas et al., 2010). Manganese and iron are co-factor and structural component of peroxidases, respectively. Therefore, these nutrients can affect this class of auxin catabolism enzymes (Campa, 1991; Fang and Kao, 2000). The appropriate management of light quality and fertilization schemes applied to mother plants, in a way to positive influence auxin biosynthesis, transport, and metabolism, could implicate in a better rooting response on subsequent cuttings produced by these stocks. The relevance of mineral nutrition for AR is highlighted by the fact that rooting phase-specific mineral nutrient compositions, optimized for cuttings themselves, have been shown to improve rooting and survival of *Eucalyptus globulus* plants (Schwambach et al., 2005). High nitrogen supply to stock plants and the resulting elevated N content in herbaceous cuttings have been shown to strongly promote AR (Druege et al., 2000, 2004; Zerche and Druege, 2009).

The initial content and composition of phenolic compounds are also transferred to cuttings from mother plants and the interaction of these metabolites with auxin and peroxidases may have effects on adventitious root formation (De Klerk et al., 1999). Flavonoids, a major class of phenolic compounds, can influence auxin transport (Peer and Murphy, 2007), mainly by interacting with efflux carrier PIN2 (PIN-FORMED 2) or affecting the distribution of other PIN proteins (Buer et al., 2010). Phenolics are also important in modulating peroxidase activity and could also act as antioxidants, preventing auxin degradation at cutting bases (De Klerk et al., 1999).

Several investigations have pointed out that the initial carbohydrate content of the cutting, should be enough to supply the energy reserves throughout the rooting period (Veierskov, 1988; Husen, 2008). On the other hand, there is evidence that carbohydrate allocation and distribution within the cutting could be more important than the content itself (Druege et al., 2000; Druege, 2009; Ruedell et al., 2013). Light and current photosynthesis of cuttings could play an important role in this scenario, influencing carbohydrate metabolism and reallocation (Hoad and Leakey, 1996; Rapaka et al., 2005). In the rooting recalcitrant *E. globulus*, donor plants grown in medium devoid of sugar and exposed to white irradiance promoted AR in cuttings, whereas presence of exogenous sugar in donor plant media favored rooting in the easy-to-root *E. saligna*, with no significant effects of irradiance (Corrêa et al., 2005). Appropriate light environment applied to mother plants may increase carbohydrate sink capacity at the root formation site in cuttings derived therefrom.

Maturation negatively affects the regenerative ability of plant material and, as a consequence, diminishes its AR potential. The content and profile of phenolic compounds, as well as the contents of carbohydrates and auxins, switch according to maturation state, correlating with rooting competence (Fernández-Lorenzo et al., 2005; Husen and Pal, 2007; Osterc et al., 2009). The use of juvenile-like material can help overcoming this limitation (Cameron et al., 2003; Kibbler et al., 2004). In vegetative propagation of trees, the use of minicutting technique, both in hydroponic or sand bed minihedges, affords a better environmental control of ministumps (mother plants), improving their physiological quality, and, consequently, the rooting propensity of the minicuttings obtained (Assis et al., 2004; Schwambach et al., 2008).

The molecular basis of rooting competence is an essential aspect of AR. In principle, even if all environmental variables are ideally manipulated so as to favor AR, unless developmental competence is present, responses to the root-promoting signals do not take place and rooting fails. Developmental responsiveness is likely dependent on presence and density of functional phytohormonal receptors and signaling pathways, particularly those for auxin. A detailed investigation on AR of hypocotyls (able to root proficuously upon exposure to auxin) and epicotyls (root poorly even in presence of auxin) of 50-day old seedlings of *Pinus taeda* showed that lack of rooting responsiveness in epicotyls was not related to auxin uptake, transport, distribution among cells, or metabolism. Localized fast cell division and root meristem organization were lacking in epicotyls (Diaz-Sala et al., 1996). Application of the auxin transport inhibitor *N*-(naphthyl)phthalamic acid (NPA) up to the first 3 days after cutting severance inhibited rooting without affecting auxin concentration or metabolic status at the rooting site, suggesting a role for auxin polarity in rooting capacity that would be different than simply moving auxin to the rooting zone.

Auxin capacity to trigger gene expression has been suggested as an early and critical point in AR competence of *Pinus taeda* stem cuttings, for example (Greenwood et al., 2001). In this system, the inability to root in mature cuttings was apparently due to the lack of cells capacity to arrange themselves into root meristems in presence of auxin. Cell division and callus formation, however, occurred similarly, both in physiologically juvenile and mature cuttings, leading the authors to suggest the existence of an auxin transduction pathway specific to root meristem organization. Members of the expansin gene family are among the early auxin-induced genes during AR of pine cuttings, particularly in non-growing zones of the stem before cell divisions that result in root development (Hutchison et al., 1999). Some auxin-responsive transcription factors have been shown to play roles in the control of cell division leading to root primordia differentiation in cuttings of tree species (Sánchez et al., 2007; Solé et al., 2008; Vielba et al., 2011; Rigal et al., 2012) and are discussed in further detail in the Section “Cell Cycle and Division-New Meristems” below.

The loss of AR capacity at physiologically mature stages is often associated with the transition to flowering (phase shift from juvenile to adult stage). However, in specific organ parts or under specific culture conditions, loss of rooting capacity can take place and become easily noticeable at much earlier stages of development (e.g., seedling), providing interesting experimental systems to study this process in trees (Fett-Neto et al., 2001; Greenwood et al., 2001). Another useful model to study AR and the loss of rooting capacity is *Arabidopsis thaliana*. Using de-rooted hypocotyls of young (12 day old) and adult (26 day old) plants of the Landsberg ecotype, it was shown that AR was much slower in adult de-rooted plants and that endogenous polar auxin transport (evaluated with NPA application) was crucial for AR (Díaz-Sala et al., 2002). These authors also showed that rooting was not dependent on phase shift to reproductive phase, although a correlation was observed. The decline in rooting capacity was probably linked to age-related processes. A correlation between reduced AR capacity and flowering phase shift was also shown in detached leaves of *Arabidopsis* plants of the Columbia ecotype, but only in leaves harvested 2–3 weeks after bolting (Corrêa et al., 2012b). A possible link between flowering and AR of detached leaves was not observed by analyzing the AR kinetics in two early and two late flowering time mutants of each of two ecotypes, Antwerpen and Columbia (Corrêa et al., 2012b). Interestingly, Díaz-Sala et al. (2002) showed that AR in de-rooted hypocotyls of *Arabidopsis* adult plants depended on RGD (Arg-Gly-Asp) peptides (a family of peptides bearing this signature domain), although these were not sufficient for rooting to occur and had no effect on young plant hypocotyls. The RGD peptides may be important in causing changes to the plasma membrane of plant cells and their interaction with cell walls, perhaps affecting cytological events required for AR in adult plant hypocotyls. Taken together these data indicate that *Arabidopsis* and *in vitro* culture systems of tree species are useful tools to study developmental competence to AR.

## FEATURES ASSOCIATED WITH EXOGENOUS AUXIN SUPPLY

In addition to the already mentioned effect of endogenous auxin in adventitious root formation, it is well-established that this phytohormone can also act when exogenously supplied, entering the stem via the cut surface of cuttings. In many rooting recalcitrant species, application of exogenous auxin is needed to achieve satisfactory rooting responses (Diaz-Sala et al., 1996; Fett-Neto et al., 2001). In these cases, endogenous auxin produced in the shoot apex and transported basipetally to the cut surface may be complemented by exogenously applied phytohormone aiming at improving the rooting response (Pop et al., 2011). The absence of a shoot apical meristem has not limited AR in *Eucalyptus* microcuttings exposed to exogenous auxin (Fogaça and Fett-Neto, 2005).

Uptake of exogenously provided auxin implicates in a new auxin transport route, which enters the cuttings mostly via the cut surface (Kenney et al., 1969; Guan and De Klerk, 2000) and may be taken up by cells both through a pH trapping mechanism (Rubery and Sheldrake, 1973) and through influx carriers (Delbarre et al., 1996). Most of the supplied auxin acts at the wound site, inducing cell dedifferentiation, leading to a new root meristem later on. A portion of the supplied auxin could also be redistributed along the cutting, mostly via the xylem transpiration route (Osterc and Spethmann, 2001). In this case, auxin influx and efflux carriers would not take significant part in the process, losing directionality of the polar auxin transport throughout the plant (auxin transport is discussed ahead in detail). In fact, auxin uptake may also occur through the phloem and a better rooting performance in *Prunus subhirtella* juvenile cuttings was related to this kind of absorption path (Osterc and Stampar, 2011). However, studies with auxin transport inhibitors provided evidence that rooting in *Pinus taeda* hypocotyls is improved when exogenous auxin is incorporated in the polar auxin transport system (Diaz-Sala et al., 1996). Much of the data from different reports on interactions of exogenous auxins with the polar auxin transport system is probably difficult to compare because of the use of different auxins in the various experiments, including synthetic forms, for which the transport systems are poorly known.

## CARBOHYDRATE ALLOCATION

Carbohydrates contribute to the formation of adventitious roots by supplying energy and carbon necessary for cell divisions, establishment of the new root meristems and root formation itself. The efficient partitioning of carbohydrates between the new sink of developing roots at cutting base and the shoot meristem sink could be critical for AR (Druege, 2009). Ahkami et al. (2009) proposed that the early establishment of a carbohydrate sink at the rooting site is a key metabolic event in *Petunia hybrida* adventitious root formation. Pre-incubation of *Petunia* cuttings in the dark increased carbohydrate levels at their bases upon transfer to light, improving AR (Klopotek et al., 2010). Similarly, a higher content of soluble sugars and starch in the rooting zone were associated with higher rooting response in *Tectona grandis* cuttings (Husen and Pal, 2007). Higher accumulation of soluble carbohydrates and starch at the root formation zone in microcuttings was associated with improved rooting capacity of *E. globulus* without exogenous auxin. This condition was observed when cuttings were obtained from mother plants grown in medium devoid of sucrose and exposed for a few weeks to far-red irradiation-enriched environment (Ruedell et al., 2013). When mother plants were grown in sucrose containing medium, the positive effect of exposing stock plants to far-red enriched irradiance on microcutting rooting capacity was abolished. Inhibition of AR in carnation cuttings by high carbohydrate content has also been proposed, although the importance of establishing an auxin-stimulated carbon sink was pointed out (Agulló-Antón et al., 2011).

Growth and differentiation of tissues can be modulated by carbohydrate signals through alterations in metabolic fluxes and carbohydrate concentrations during development, which may regulate gene expression (reviewed by Rolland et al., 2006). These carbohydrate signals are generated by photosynthesis and carbon metabolism in source and sink tissues and probably play a regulatory role in adventitious root induction (Druege, 2009). Interactions between phytohormones and carbohydrates are essential part of the sugar sensing and signaling network (Rolland et al., 2006): and a glucose and auxin signaling crosstalk was shown to be important for controlling root development and growth in *Arabidopsis thaliana* seedlings (Mishra et al., 2009). Auxin supply to *Dalbergia sissoo* cuttings enhanced the content of total soluble sugars and starch, promoting AR (Husen, 2008). Different carbon sources may affect the rooting capacity of eucalypt microcuttings in a rooting phase-dependent fashion, even in absence or with suboptimal supplied auxin concentrations, particularly in the difficult-to-root *E. globulus* (Corrêa et al., 2005). Taken together, available data suggest that low carbohydrate allocation to the root formation site may limit AR. Adequate supply of these compounds is a combined function of sink strength and the capacity of the source to meet sink demand (Druege, 2009). Carbohydrates play important roles, not only by providing energy and carbon chains for biosynthetic processes in new meristems and roots, but also by affecting gene expression, in co-action with auxin.

## WOUND RESPONSE

Severance of a cutting from the donor plant has immediate consequences, including injury and the isolation from functional integrity of the whole plant condition, i.e., loss of root–shoot correlative influences (Druege, 2009). Excision of *Petunia* cuttings led to a fast and transient increase in the wound-phytohormone jasmonic acid (JA) and a continuous accumulation of soluble and insoluble carbohydrates during adventitious root formation (Ahkami et al., 2009). There is some evidence that AR is also influenced by ethylene production caused upon wounding during explant preparation, and a stimulatory role of endogenous ethylene would depend on achieving a relatively narrow concentration range (Mensuali-Sodi et al., 1995). In fact, for some *in vitro* studies, the use of anti-ethylene chemicals has resulted in improved rooting responses (De Klerk et al., 1999).

Adventitious rooting in cuttings may be compared to a stress-induced reprogramming of shoot cell fate. Acclimation to stress is often accompanied by metabolic re-adjustment. The alternative oxidase (AOX) plays a central role in determining reactive oxygen species equilibrium in plants and can be induced in response to diverse abiotic and biotic stress conditions (Santos-Macedo et al., 2012). Secondary metabolism during AR may be associated with AOX activity. Phenylpropanoid derivatives, especially phenolic acids and lignin, are known to be closely related to the regulation of cell division and differentiation. Enhanced accumulation of phenolic acids and some flavonoids was found to correlate with *in vitro* rooting (De Klerk et al., 1999). Moreover, a complex interaction between AOX and H_2_O_2_ signaling is apparent. Application of H_2_O_2_ could replace added auxin as a rooting agent in olive cuttings (Santos-Macedo et al., 2009) and the presence of an AOX inhibitor, salicylhydroxamic acid (SHAM), reduced rooting even in presence of exogenous auxin (Santos-Macedo et al., 2012).

Phenolic compounds are known to protect plants from oxidative stress (Jaleel et al., 2009) and allow the containment of excessive wound response that may inhibit subsequent regeneration processes (De Klerk et al., 2011). Phloroglucinol and ferulic acid displayed antioxidant action, protecting IAA from decarboxylation and the tissue from oxidative stress in *Malus* “Jork 9,” thereby promoting AR. The decarboxylation was attributed to the wound response and did not occur to such an extent in non-wounded plant tissues. The action of the phenolic compounds suggests that, at least in part, rooting depends on the inhibition of IAA decarboxylation caused by wounding, so that more auxin is available to induce roots (De Klerk et al., 2011).

Hydrogen peroxide, a form of reactive oxygen, functions as a signaling molecule that mediates various physiological and biochemical processes, as well as controls responses to various stimuli in plants (Neil et al., 2002). Li et al. (2009b) showed that H_2_O_2_ might function as a signaling molecule involved in the formation and development of adventitious roots in mung bean seedlings. Production of H_2_O_2_ was markedly induced in indole-3-butyric acid (IBA)-treated seedlings suggesting that IBA induced overproduction of H_2_O_2_ and promoted AR via a pathway involving H_2_O_2_. In another study, Li et al. (2009c) suggested that the mechanism underlying the IBA and H_2_O_2_-mediated facilitation of adventitious root formation is the early decrease of peroxidase and ascorbate peroxidase activities in IBA and H_2_O_2_-treated seedlings. The decrease in activity of these enzymes would be relevant to generate the necessary high level of auxin and H_2_O_2_ required for adventitious root induction.

## WATER RELATIONS

The availability of water is one of the most important factors favoring root development, as cuttings have to maintain a positive water balance while roots develop (Loach, 1988). Puri and Thompson (2003) carried out a study to examine the influence of three levels of initial water potential in stem cuttings of *Populus* (dried, soaked, and fresh) on plant water status and rooting capacity under controlled environmental conditions, in combination with planting in soils with different water potential. Results clearly showed that soil moisture had a major effect on rooting. Water-stressed cuttings took a longer time to root and formed fewer roots. Pre-soaking of cuttings had a positive effect on rooting, mainly under the drier soil moisture conditions. Although unrooted hardwood cuttings needed moister soil to initiate rooting, once roots were established, they could tolerate somewhat drier conditions. In good agreement, cutting survival and AR were highest in moister substrate for stem cuttings of juniper (*Juniperus horizontalis*), azalea (*Rhododendron*), and holly (*Ilex crenata*; Rein et al., 1991).

Gas exchange and water relations have also been simultaneously evaluated. Relative water content (RWC) of leaves and osmotic potential increased upon formation of root primordia in *Poinsettia* cuttings (Svenson et al., 1995). Following formation of root primordia, and concurrent with increasing RWC and osmotic potential, stomatal conductance (g) increased. As roots initially emerged, net photosynthesis and g increased rapidly and continued to increase with further root primordia development and subsequent emergence of adventitious roots. Abscisic acid (ABA) often accumulates under water stress conditions and is a known inhibitor of cell cycle progression (Wolters and Jürgens, 2009). Hence, the level of water stress is a relevant factor for cutting establishment that should be minimized in order to avoid losses and slow establishment of plants.

## PHYTOHORMONAL BALANCES: THE SEESAW OF PROMOTION VERSUS REPRESSION

Auxins have a rhizogenic action during the root induction phase (generally from cutting severance up to 96 h) and stimulate cells at the cutting base to engage in the establishment of meristemoids (Garrido et al., 2002). The same phytohormones become inhibitory after 96 h and may arrest or inhibit growth of root primordia (De Klerk et al., 1999). Diaz-Sala et al. (1996), using NPA treatments, showed that the initial 48 h were crucial for auxin-dependent root induction in pine. In addition, mRNA levels of transcription factors possibly related to root meristem fate, as well as cell wall remodeling genes, were increased in presence of exogenous auxin at 24 h (Hutchison et al., 1999; Sánchez et al., 2007; Solé et al., 2008; Vielba et al., 2011).

In general, free IAA endogenous levels have a transient increase during the induction phase, pass through a minimum at the initiation step and resume an increase in the expression phase (Bellamine et al., 1998). The importance of auxin at the induction and expression phases (first and last steps) of the rooting process was demonstrated through the use of anti-auxins, which prevent auxin from exerting its functions. In poplar cuttings, anti-auxins present at one of these phases caused significant inhibition of AR (Bellamine et al., 1998). Moreover, Negishi et al. (2011) compared easy and difficult-to-root lines of *E. globulus* and verified that IAA level was twofold higher in the easy rooting line, confirming the importance of IAA in AR.

A screen for chemicals that cause inhibition of cytochrome P-450 identified one chemical, MA65, which led to an increase in the number of roots of *Arabidopsis* seedlings and twofold higher IAA levels compared to the untreated *Arabidopsis* (Negishi et al., 2011). The observed phenotype was similar to the mutant *superrot2* (*sur2*) which contains high concentrations of free IAA (Delarue et al., 1998) due to a defect in the *SUR2* gene, which encodes the CYP83B1 protein, a cytochrome P450-dependent monooxygenase (Barlier et al., 2000). This increase in IAA production probably happens because cytochrome P450 inhibition blocks the synthesis of indole glucosinolates, providing more substrate (indole-3-acetaldoxime) for the biosynthesis of IAA (reviewed by Bak et al., 2001). The same chemical MA65 was effective for inducing AR in *E. globulus,* but the exact mechanism of action of the chemical in this species awaits further investigation.

The regulation of auxin levels can be done by conjugation of excessive auxin to inactive forms, preventing phytohormone accumulation in the tissue. Auxin degradation, e.g., by peroxidases, is another means of controlling the activity of these regulators. Auxins of different metabolic lability may be conjugated: high stability 1-naphthalene acetic acid (NAA), low stability IAA and moderate stability IBA (De Klerk et al., 1999). IAA can form conjugates with sugars, amino acids, and peptides and these forms are considered resistant to oxidases. IAA can be stored in higher plants as IAA conjugates which might be hydrolyzed depending on the plant demand for free auxin; IBA can also yield IAA by β-oxidation (Woodward and Bartel, 2005). Even if in some cases the conjugation process can be irreversibly inactivated by oxidation (Epstein and Ludwig-Müller, 1993), the most part of auxin conjugates are reversible (De Klerk et al., 1999). When IAA and IBA were exogenously applied to cuttings of *Pisum sativum* L. during adventitious root formation, conjugation of auxins with aspartic acid was the predominant route of metabolism, forming indole-3-acetylaspartic acid (IAAsp) and indole-3-butyrylaspartic acid (Nordström et al., 1991). The authors also verified that the levels of IBA remained high for longer time than those of IAA, indicating higher stability of IBA in rooting solution.

Some gene members of the *GH3* family are involved in the maintenance of auxin homeostasis, contributing to regulation of the auxin pool (Staswick et al., 2005; Chapman and Estelle, 2009). *GH3* genes encode IAA-amide synthetases, which act in the conjugation of physiologically active free IAA excess to amino acids (Staswick et al., 2005). In the moss *Physcomitrella patens*, knock-out of *GH3* genes increased the sensitivity to auxin causing growth inhibition (Ludwig-Müller et al., 2009). Altered auxin sensitivity was also observed in *Arabidopsis* *thaliana* by overexpression and insertional mutation of *GH3* genes (Staswick et al., 2005). Gutierrez et al. (2012) reported a crosstalk of IAA and JA in which AR-inhibitory JA levels are reduced by conjugation with amino acids through expression of *GH3.3*, *GH3.5*, and *GH3.6* auxin-induced genes, via the action of *ARF6* and *ARF8*, leading to increased number of adventitious roots. GH3 genes would be required for fine-tuning adventitious root initiation in the *Arabidopsis thaliana* hypocotyl, where JA homeostasis is under auxin control (Gutierrez et al., 2012). Curiously, JA accumulation at the cutting base has been shown to be an early, transient, and critical event for rooting of *Petunia* cuttings, and has been discussed to contribute to increasing cell wall invertases and sink strength at the cutting base (Ahkami et al., 2009). Brassinosteroids (BR) have been shown to exert a mild negative regulation of JA-induced inhibition of root growth (Huang et al., 2010). If this applies to AR as well, there could be an additional antagonist crosstalk between JA and BR, regulating the formation phase.

Cytokinins and ethylene have an overall inhibitory effect on induction, but can play a promotive effect during the first 24 h, when cytokinins start to drive cell cycle movement, culminating in mitotic processes (De Klerk et al., 1999; De Klerk, 2002), and ethylene may contribute to auxin transport regulation (Lewis et al., 2011) or to increase the number of auxin-responsive cells (De Klerk and Hanecakova, 2008). Corrêa et al. (2005) observed that kinetin inhibited AR if present during the induction phase in *E. globulus*. The cytokinin type-B response regulator PtRR13, a transcription factor that acts as positive regulator in the cytokinin signaling pathway, has been shown to negatively regulate AR in *Populus*; PtRR13 inactivation upon cutting severance due to the removal of root sources of cytokinin, would alleviate AR inhibition, allowing basipetally transported auxin to accumulate at cutting base, promoting AR (Ramírez-Carvajal et al., 2009). Ethylene has been shown to promote adventitious root and inhibit lateral root development, predominantly by affecting auxin transport in distinct ways (Negi et al., 2010). Lateral root development inhibition by ethylene was linked to increased expression of PIN3 and PIN7 and auxin transport, preventing auxin accumulation maxima required for pericycle cell activation in roots; in contrast, adventitious root stimulation by ethylene in shoots was due to reduced auxin transport in these organs, favoring auxin accumulation and AR (Lewis et al., 2011). Stimulation of AR in flooded tomato plants was dependent on ethylene accumulation followed by auxin transport increase and allocation to flooded parts of the stem base. Local accumulation of auxin can cause further ethylene production, enhancing the process (Vidoz et al., 2010).

Strigolactones are also involved in adventitious root formation, mostly as repressors, by inhibiting the first divisions of founder cells independently of cytokinins, and perhaps negatively regulating basipetal auxin movement in *Arabidopsis thaliana* and pea (Rasmussen et al., 2012). Upon cutting severance, the content of strigolactones would reduce, since roots are a major source of these phytohormones.

Nitric oxide (NO) has also been proposed as a player in the control of AR. In cucumber, AR was favored by NO, acting downstream of auxin, possibly through different transduction pathways (Lanteri et al., 2009). Auxin-stimulated NO production would increase phosphate cyclic nucleotides cGMP (cyclic guanosine monophosphate) and cADPR (cyclic adenosine 5^′^-diphosphate ribose), triggering activation of Ca^2+^ channels in the plasmalemma. The release of phospholipids promoted by NO would provide substrates for phospholipases, whose activity and released products could further activate Ca^2+^ release to the cytosol and activate both calcium-dependent protein kinases (CADPKs) and mitogen-associated protein kinases (MAPK). These kinases would in turn lead to cell growth and differentiation associated with AR. NO-promoted AR was also reported for other species, including greenhouse–grown cypress (Lanteri et al., 2009) and *E.* *grandis* (Abu-Abied et al., 2012). In these studies a co-action of NO and auxin has often become apparent, with NO being induced by auxin. In sunflower, it was suggested that NO could participate with auxin in adventitious root initiation and expression (extension), whereas induction would depend only on auxin (Yadav et al., 2010). Studies on AR of *Tagetes erecta* (marigold; Liao et al., 2009), *Vigna radiata* (mung bean; Li and Xue, 2010), and *Chrysanthemum* (Liao et al., 2010) have suggested that H_2_O_2_ and NO may act together, possibly as parallel independent pathways dependent on Ca^2+^, converging on the activation of MAPK cascades leading to AR. A novel interaction of NO and auxin has been shown at the level of NO dependent *S*-nitrosylation of TIR1 auxin receptor, enhancing TIR1-Aux/IAA binding and degradation of the latter, promoting auxin-mediated gene expression (Terrile et al., 2012). The extent of this interesting mechanism in the context of AR is a key research topic to be explored.

Gibberellins (GAs) are generally considered inhibitors of AR. This has been shown, for example, in poplar (Busov et al., 2006). Moreover, lateral root number and growth were promoted in plants with defects in GA production or perception, so that higher root mass and highly branched roots were produced. This inhibitory effect of GA on lateral root development has been partially attributed to changes in polar auxin transport (Gou et al., 2010). In contrast, initiation and elongation of adventitious roots was promoted by GA in deep water rice (Steffens et al., 2006). It is possible that GA may have an AR phase-dependent effect, being inhibitory to root induction and stimulatory to formation. ABA also acts as an inhibitor of lateral root development in *Arachis hypogaea* by blocking cell cycle progression (Guo et al., 2012). Inhibition of adventitious root formation step by ABA was also reported in deep water rice (Steffens et al., 2006).

Polyamines are nitrogen containing, polycationic, low molecular weight aliphatic compounds that can be found in meristematic and actively growing tissues. These metabolites (e.g., putrescine, spermidine, spermine) play various roles, mostly related to control of cell division, development, and stress responses. Because of their positive charges, polyamines are capable of binding to nucleic acids, proteins, and membranes, therefore potentially being able to interfere in processes such as gene expression, cell signaling, membrane stabilization, and modulation of some ion channels (Kusano et al., 2008). Polyamines have been treated as biochemical markers of AR because their concentration peak is consistently associated with the end of the induction phase, similar to auxins. In various unrelated species, AR or promptness to develop adventitious roots is often observed when polyamines peak at the end of adventitious root induction and are metabolized before or at the formation phase (Neves et al., 2002; Arena et al., 2003; Naija et al., 2008).

**FIGURE 1:**
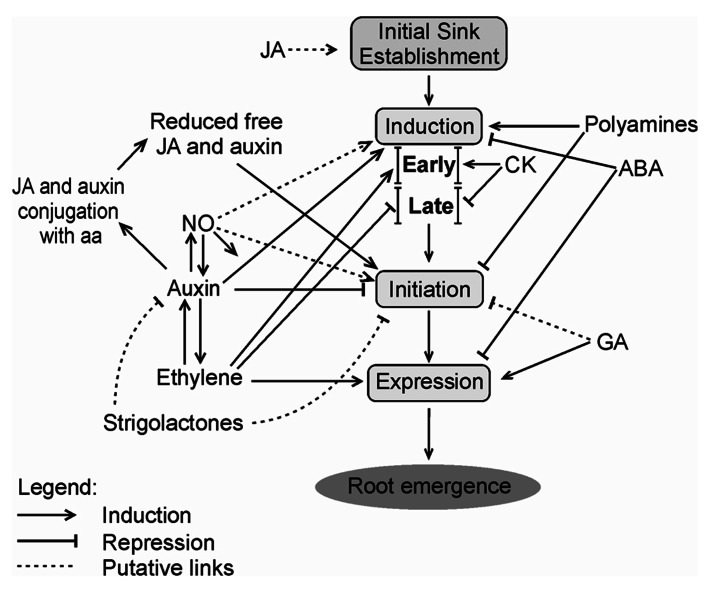
**Possible phytohormonal interactions during distinct phases of the adventitious rooting process**. JA may promote initial carbohydrate sink establishment before induction or at its early moments. Induction phase is positively regulated by auxin, polyamines and, in early stage, by CK and ethylene. However, during late induction, cytokinin and ethylene act as negative regulators. ABA has a negative effect on AR induction. Initiation phase is inhibited by auxin, polyamines, and GA. JA and auxin are conjugated with aa, so the levels of these phytohormones decrease allowing the progress of the initiation phase. Strigolactones may repress auxin action by reducing its transport and accumulation, or may directly inhibit AR. In contrast, NO is regarded as a stimulator of AR, both during induction and initiation phases. Ethylene increases auxin transport, stimulates expression, but shows a direct repressor effect at induction phase, except perhaps at its early stage, as pointed out above. Auxin may also promote ethylene biosynthesis. Expression phase is induced by ethylene and GA, and suffers repression of ABA. Root emergence is the visible phenotype after the expression phase. Relative positions of phytohormone names within the scheme are not meant to represent differences in importance, but aim at better clarity of the layout. JA, Jasmonic acid; CK, cytokinin; ABA, abscisic acid; GA, gibberellin; NO, nitric oxide; aa, amino acids.

A tentative model summarizing some of the main data on phytohormonal control of AR is shown in **Figure 1**.

Given the importance of phytohormones, particularly auxins, to the control of AR, the next three sections will examine fundamental aspects of cell cycle control, root tissue differentiation, auxin transport, metabolism, and action. However, it must be emphasized that most of the knowledge presented in these sections is derived from investigations directed to general plant development or development of primary or lateral roots. Although it is clear that these processes are important in AR, their exact contribution in the specific context of the process is far from complete.

## CELL CYCLE AND DIVISION – NEW MERISTEMS

Cell divisions in meristems depend on the cell cycle, which involves a mechanism governed by cyclin-dependent kinases (CDKs; Inzé and Veylder, 2006; De Veylder et al., 2007). The association of CDKs and cyclins is required for the induction of cell cycle progression, through phosphorylation of substrates at the transition points of some of its phases (Inzé and Veylder, 2006). G1–S transition is regulated by D-type cyclins (CYCD), which might also be involved in G2–M transition. A-type cyclins (CYCA) are present in S–M, whereas B-type cyclin (CYCB) act in G2–M transition and during M period (De Veylder et al., 2007). G1–S transition may be blocked by abscisic acid, causing inhibition of lateral root primordia initiation in peanut (Guo et al., 2012).

Cyclin-dependent kinases present in plants are A-type (CDKA) and B-type (CDKB), the latter being plant-specific. CDKB accumulation depends on the cell cycle period, specifically the B1 subclass in the S phase and after G2 until mid-M and B2 subclass, reaching a peak in G2 and M (Boudolf et al., 2006; De Veylder et al., 2007). Moreover, a plant homolog of the tumor suppressor Retinoblastoma (pRb), the RETINOBLASTOMA-RELATED (*RBR*) gene is considered a key cell cycle regulator downstream of the SCARECROW (*SCR*) patterning gene, a member of the GRAS family of transcription factors, acting in the control of cell division, differentiation and cell homeostasis (Wildwater et al., 2005; Borghi et al., 2010). The transcription factors *E2F* and *MYB3R* also take part in the cell cycle control, involved in activation/inactivation of the S-phase and M-phase genes, respectively (De Veylder et al., 2007). Cytokinin and auxin are the main hormones involved with cell proliferation and are indispensable for the progression of the cell cycle (Dewitte and Murray, 2003).

Plant development depends on meristem growth, which happens when cell division predominates over differentiation. The root meristem size is controlled by the balance between cell division and differentiation, where cytokinins and auxins act antagonistically and play important roles (Dello Ioio et al., 2007, 2008; Moubayidin et al., 2009, 2010). In *Arabidopsis*, the *short hypocotyl 2* (*SHY2*) gene acts as a negative regulator of auxin signaling (Tian et al., 2002) by forming heterodimers with *ARF* transcription factors and thus avoiding the activation of auxin-responsive genes. *SHY2* expression is activated by the presence of cytokinins via the route of AHK3 (*Arabidopsis* histidine kinase 3) receptor kinase/cytokinin-responsive *ARR1* transcription factor, and leads to negative regulation of *PIN* genes, involved in the efflux of auxin, which consequently causes a reduction in the root meristem size (Dello Ioio et al., 2008). Moreover, auxins can cause *SHY2* degradation, and promote the expression of *PIN* genes (Dello Ioio et al., 2008). Furthermore, the transcription factor *ARR12* and GAs also seem to participate in this regulation, *ARR12* inducing a low level of *SHY2* expression and GAs repressing expression of *ARR1* during post-germination meristem growth (Moubayidin et al., 2010).

The root apical meristem is composed of sets of self-renewing and undifferentiated stem cells that allow continued root growth. The quiescent center (QC) takes part in maintaining this condition by supporting meristematic identity of the initial cells around it (Van den Berg et al., 1997; Osmont et al., 2007; Arnaud et al., 2010). The QC cells form part of a region that has a low rate of mitosis, and are histologically distinct from neighboring cells (Doerner, 1998). QC serves as a reservoir of cells for regeneration and ensures the persistence of the apex meristem, as they have self-renewal and self-maintenance capacities. Hormonal activity is important for the QC maintenance and organization (Sabatini et al., 1999; Ortega-Martínez et al., 2007). Reporter genes fused to promoters regulated by auxin were visualized with maximum expression in the position of the QC and root columella (Sabatini et al., 1999). Data obtained by Ortega-Martínez et al. (2007) suggest that ethylene promotes cell division in the QC, indicating that auxin alone would not be sufficient to carry out this function. Surrounding QC, initial cells perform stem cell-like divisions to generate a new initial and a daughter cell, so that the meristem gives rise to all different cell types (Van den Berg et al., 1997).

Some transcription factors, such as *SCR* and *SHORTROOT* (*SHR*), also belonging to the GRAS family of proteins, have crucial role in maintaining the meristematic cells pluripotent identity (Sabatini et al., 2003; Cui et al., 2007). *PLETHORA1* (*PLT1*) and *PLETHORA2* (*PLT2*) are also involved with meristem maintenance, are induced by auxin, and act in parallel with *SHR* and *SCR*, encoding transcription factors *AP2*-like (Aida et al., 2004). *SCR* expression appears to depend of the gene *PDR2* (Ticconi et al., 2009), acting indirectly on QC maintenance. The distribution of *PLT* mRNA is associated with the peak of auxin in stem cells and QC in root meristem (Sabatini et al., 1999). The homeobox transcription factor *WOX5* (*WUSCHEL-RELATED HOMEOBOX 5*), root homologue of the shoot *WUSCHEL* (*WUS*), also has a function in the stem cell maintenance and signaling (Sarkar et al., 2007; Miwa et al., 2009; Stahl et al., 2009).

The involvement of some of these transcription factors in AR in cuttings of tree species has been described. An approach based on cDNA subtractive libraries from rooting competent cuttings of *Pinus radiata* and *Castanea sativa* treated or not with exogenous auxin (Sánchez et al., 2007) yielded data supporting the involvement of clones with homology to *SCR* (*SCR*-like or *SCL*). The content of the corresponding mRNA of these genes increased in both species upon auxin exposure within the first 24 h of the rooting process, coinciding with cell reorganization preceding divisions and establishment of defined root primordia. In *Pinus radiata*, an *SHR*-related clone was identified with an expression pattern similar to that of *SCL*, except for the fact that it was auxin-independent, possibly playing a role in root meristem formation and maintenance, as well as in the cambium zone of hypocotyls (Solé et al., 2008). The expression of *SCL* in *C. sativa* cuttings of juvenile and mature stages was examined in detail (Vielba et al., 2011). A combination of quantitative real time polymerase chain reaction (PCR) and *in situ* hybridization showed that *CsSCL1* was upregulated by auxin, localizing more strongly in the cambium layer and derivative cells in rooting competent shoots, whereas for root incompetent shoots its signal was more diffuse and evenly distributed in the phloem and parenchyma (Vielba et al., 2011). The authors suggest that *CsSCL1* may determine which cells will engage in the root differentiation route, athough they observed that expression of this gene was also present in lateral roots and axillary buds.

Recently, *AINTEGUMENTA LIKE1* (*PtAIL1*), a member of the *AP2* family of transcription factors, has been shown to be associated with cell division and further establishment of adventitious root primordia in *Populus trichocarpa* (Rigal et al., 2012). Transgenic poplar overexpressing *PtAIL1* displayed higher number of adventitious roots, whereas RNA interference (RNAi) downregulation of the same gene transcript resulted in delayed AR. A number of genes were co-regulated with *PtAIL1* based on microarray and comparative analyses of modified poplar lines up or downregulated for the *AP2* transcription factor, included among these additional transcription factors, such as *AGAMOUS-Like6* and *MYB36* (Rigal et al., 2012).

## THE CENTRAL ROLE OF AUXINS: TRANSPORT, CONTROL OF LOCAL CONCENTRATION, TIMING, AND METABOLIC DYNAMICS

Auxins are very important for determining pattern in plants. Their spatial distribution is determinant for proper formation of the axis along the plant body. Auxin transport has two main forms: (a) rapid (up to 10 cm per h), often referred to as non-polar, bidirectional transport in the phloem sieve elements, (b) slow (approximately 10 mm per h) or polar, mediated by transporters (Kerr and Bennett, 2007), mostly in vascular parenchyma. Rapid transport in the phloem conducting cells essentially obeys source–sink relations and involves both free IAA and inactive conjugates (Friml and Palme, 2002). Studies with radiolabeled IAA applied to pea leaves indicated that both transport pathways may communicate, at least from the non-polar to the polar system (Cambridge and Morris, 1996). There is also evidence that phloem-based transport may become relatively more important than polar transport, at least in roots, at later stages of seedling development (Ljung et al., 2005).

The polar transport of the major endogenous auxin IAA has specific carriers, which allow intercellular auxin flow and are well-known in *Arabidopsis*. In stems, the transport is active, polar, and basipetal. According to the chemiosmotic model (Raven, 1975), there is a pH gradient between the intra- and extracellular medium, generated by the action of proton pumps in the plasma membrane, which drive protons into the apoplast, making it acidic. In the apoplast, IAA can be found both in anionic and protonated forms, the latter being more lipophilic and capable of easily diffusing through the plasma membrane (Woodward and Bartel, 2005; Zazimalová et al., 2010). On the other hand, the anionic form lacks this capacity and, for it to enter the cell, the action of auxin influx carriers is required. These carriers are amino acid permease-like proteins of the AUX1/LAX family (reviewed in Vieten et al., 2007). These proteins act as H^+^/IAA^-^ symporters and may participate in lateral root emergence and root hair development (reviewed by Vanneste and Friml, 2009).

Members of the PIN Formed (PIN) protein family are involved with auxin efflux and their asymmetric distribution in the cells is fundamental to the characteristic polar basipetal transport along the stems. The correct localization of PIN proteins is determined by its phosphorylation status, defined by the balance between the kinase protein PID (PINOID) and the phosphatase PP2A. In the case of emerging primordia, the expression of PID is activated, turning PIN protein to a phosphorylated form, leading to its apical localization in the cell. On the other hand, in most situations, PP2A is more active than PID, leading to dephosphorylated PIN protein, resulting in a basal localization in the cell (Michniewicz et al., 2007). Furthermore, the NPA-binding protein and actin filaments of the cytoskeleton also function in the correct positioning of the PIN proteins (Muday and DeLong, 2001). This family of transmembrane proteins has eight members in *Arabidopsis* which are considerably homologous and functionally redundant, being involved in tropisms, embryo development, root meristem patterning, organogenesis, and vascular tissue differentiation (reviewed by Krogan and Berleth, 2007 and Vanneste and Friml, 2009). The Multidrug/P-glycoproteins of the ABCB (ATP-binding cassette B) transporter family (ABCB/MDR/PGP) also contribute to auxin transport, being more closely related to non-polar auxin efflux and maintenance of the main auxin fluxes (Geisler and Murphy, 2006). These transporters may also play a possible role in short-distance lateral auxin movement.

Basipetal auxin transport is also affected by the red/far-red (R:FR) light ratio (Morelli and Ruberti, 2002). In open daylight (high R:FR), auxin moves from the shoot to the root mainly through the central cylinder. However, in shade conditions (low R:FR), a new route, by the outer cell layers, is preferred. This alternative route is less effective and leads to increase in auxin levels in cell layers external to the central cylinder in the stem, enhancing cell elongation in this organ. Consequently, less auxin is transported through the vascular system, decreasing vascular differentiation and the auxin content reaching the root.

Recent findings revealed the function of a new family of putative auxin transporters, the PIN-LIKES (PILS; Barbez et al., 2012; Feraru et al., 2012). These proteins are considered evolutionarily older than PIN proteins and probably preceded the PIN-dependent auxin transport (Feraru et al., 2012), but are similar to PIN family members and also contain the auxin transport domain, predicted to carry out this function. The PILS proteins are localized in the endoplasmic reticulum (ER) and are involved in the intracellular transport of free IAA from cytosol to ER (Barbez et al., 2012; Feraru et al., 2012). According to these findings, PILS activity promotes auxin accumulation in the ER by increasing amide auxin conjugates, reducing free auxin levels. This action could be involved in a compartmentalized-type regulation of auxin metabolism (Barbez et al., 2012). The PIN family member PIN5, which is localized in the ER, is also suggested as an intracellular auxin carrier, stimulating the formation of auxin amino and ester conjugates and their transport to the ER (Barbez and Kleine-Vehn, 2012).

Auxin amino acid and glucose conjugates can also be stored in the vacuole (Ueda et al., 2011). The transport into this cellular compartment has been suggested as an action of the ABC transporter AtMRP5 (*Arabidopsis thaliana* multidrug resistance 5). *Atmrp5-1* mutants, defective in *MRP5* expression, have shown higher free auxin levels and inhibition of root elongation (Gaedeke et al., 2001). This could be due to increased levels of free auxin in the cytoplasm of root cells caused by a disruption in moving auxin conjugates away from the cytoplasm.

Considering other auxins, such as the endogenous IBA and the synthetic auxin (NAA), relatively little is known about transport and metabolism. IBA is more stable than IAA and persists for longer in plant tissues (De Klerk et al., 1999), being basipetally transported in seedling hypocotyls (Rashotte et al., 2003), similarly to IAA. However, IBA seems not to be transported in inflorescences, unlike IAA (Rashotte et al., 2003). Mutations affecting IAA transport did not cause significant effects in IBA transport. The differences between IBA and IAA transport suggest that IBA might use distinct transporters from those used to move IAA (Strader and Bartel, 2011). NAA is more stable than the above auxins and is probably transported by different carriers, as revealed by *aux1* loss-of-function mutants, which respond normally to NAA (Yang et al., 2006).

The formation of auxin gradients, originated by the combined processes of biosynthesis, conjugation, and degradation, as well as inter- and intracellular transport, independently of type, is relevant for both plant morphogenesis and determination of tissue patterns (Vanneste and Friml, 2009; Overvoorde et al., 2010; Simon and Petrasek, 2011). Previous studies of PIN expression and auxin distribution in *pin* mutants showed that PIN proteins are the major players in directional distribution networks that mediate auxin maxima and gradients during different developmental processes (reviewed by Vieten et al., 2007). In the developing embryo, the localization of PIN proteins assumes positions of auxin accumulation along the stages of development and form auxin convergence points, necessary for cotyledon initiation and positioning at the late globular stage (reviewed by Krogan and Berleth, 2007). In shoot apical meristems, auxin promotes *PIN1* expression, which generates auxin accumulation at the sites of leaf primordia formation. These, once established, promote a drain of auxin, which will accumulate at a certain distance from the early primordia, enabling the phyllotactic pattern to be established (reviewed by Berleth et al., 2007).

Recent evidence points to a possible role of APY (apyrases) in regulating auxin transport (Liu et al., 2012). Exogenous ATP is capable of inhibiting auxin transport and gravitropic response in *Arabidopsis*. Apyrases (triphosphate diphosphohydrolases) are enzymes that participate in limiting ATP content. Polar IAA transport in roots and hypocotyls was reduced in *apy2* null mutants when these were suppressed of *APY1* (apyrase 1) expression by an estradiol-induced RNAi. Basal portions of APY-suppressed hypocotyls accumulated less free IAA and morphological defects were seen in roots with the same genetic modification. Problems in gravitropic asymmetry of auxin content were detected by means of DR5::GFP constructs in APY reduced plants, either genetically or treated with APY chemical inhibitors. The relevance of apyrase participation in auxin transport during AR is presently unclear and should be object of further investigation.

Auxin gradients are also very important for root organogenesis and both primary and lateral root formation are issues that had good advances in the last decades. Studying root outgrowth in *Arabidopsis*, Blilou et al. (2005) concluded that PIN-mediated modulation of auxin distribution controls both cell division and elongation, affecting meristem, elongation zone, and final cell sizes. Dubrovsky et al. (2008) revealed a spatial and temporal correlation of auxin maxima with developmental reprogramming, resulting in lateral root initiation (LRI). The sites and frequency of LRI are controlled by variations in auxin concentration in pericycle cells, which might be correlated with changes in PIN protein localization upon gravistimulation (Benkova and Bielach, 2010). These events will culminate in lateral root primordia formation.

Genetic studies revealed that *pils2pils5* double loss of function mutant had higher free auxin levels, increased hypocotyl growth and presence of lateral roots, which were longer and more abundant than in the PILS5 gain of function phenotype. This evidence suggests that PILS2 and PILS5 could have specific functions in the cellular regulation of root growth (Barbez et al., 2012).

**FIGURE 2:**
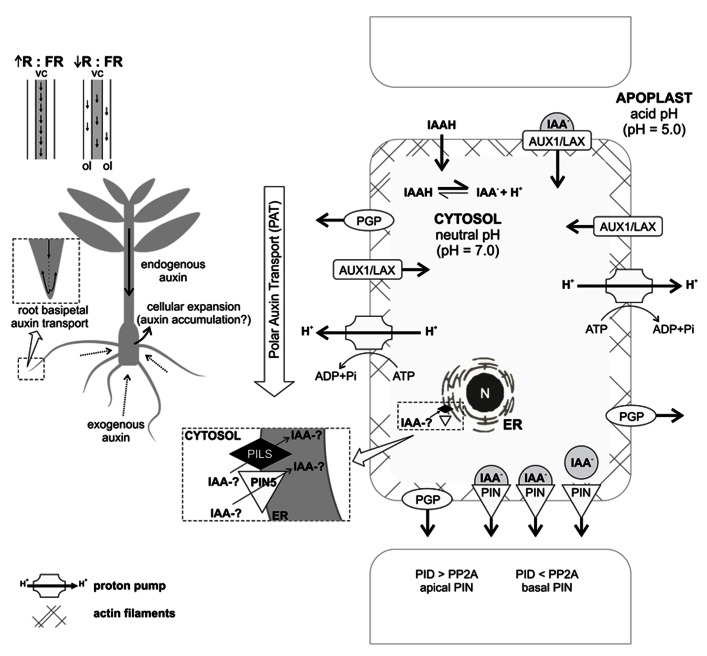
**Concept of auxin transport processes probably involved in AR**. Active, polar, and basipetal auxin transport contributes to auxin accumulation in the rooting zone of a cutting. Endogenous auxin is transported through the stem in an active, polar, and basipetal way. In daylight, when there is high red:far-red ratio (R:FR), the transport is mainly through the central cylinder (vc). In shade conditions, with low R:FR ratio, a less efficient route by the outer cell layers (ol), is preferred. When exogenous auxin is applied to the medium, it is absorbed by diffusion, which can cause cellular expansion in the basal part of the plant, perhaps due to auxin accumulation. Once adventitious roots are established, they provide a follow up to stem basipetal transport, continuing through the stele as acropetal transport in roots and then basipetal through the subepidermal cell layers of the newly formed organs. Intercellular auxin transport involves specific carriers: AUX1/LAX proteins, related with auxin influx; PGP proteins, related with auxin efflux and lateral transport; and PIN proteins, which have an asymmetrical distribution and allow directed auxin efflux. The PIN correct localization in the cell and the route of the auxin flow is determined by the balance between the kinase protein PID and the phosphatase PP2A. Concerning intracellular transport, auxin can be transported into the endoplasmic reticulum (ER) by the action of PILS proteins and PIN5, which reduce free auxin levels and increase auxin conjugates. It remains to be elucidated if auxin conjugates can be formed into the ER or just in cytosol. For more details, see text. N – nucleus; IAA-? – auxin in free or conjugated form.

However, relatively little is known about the effects of polar and non-polar auxin transport during adventitious root formation. Using inhibitors of polar auxin transport, various investigations in cuttings or de-rooted seedlings have provided evidence for a significant contribution of this type of transport to AR (e.g., Nordström and Eliasson, 1991; Liu and Reid, 1992; Koukourikou-Petridou and Bangerth, 1997; Guerrero et al., 1999; Garrido et al., 2002; Nicolás et al., 2004). Few studies analyzing the expression of genes encoding auxin carriers during adventitious rhizogenesis were conducted in de-rooted pine seedlings (Brinker et al., 2004), intact rice plants (Xu et al., 2005), carnation cuttings (Oliveros-Valenzuela et al., 2008; Acosta et al., 2009), and mango cotyledon segments (Li et al., 2012). The studies with carnation and mango showed the requirement of increased expression of auxin transporters and increase of polar auxin transport during the induction and formation phase of AR. However, in the case of pine seedling cuttings, increased expression was linked to root formation (Brinker et al., 2004). In rice, the expression of *OsPIN1* was also important during root formation (Xu et al., 2005). Taken together, these findings corroborate the role of auxin in controlling organogenesis, but more studies are necessary to clarify the effects of auxin carriers in AR, mainly in woody species. A summary of mechanisms and factors possibly contributing to transport and local concentration of auxin during AR is illustrated in **Figure 2**.

Considering cuttings used for vegetative propagation, the progressive accumulation and local concentration of auxin in the base of the cuttings seems to be important to generate the peak necessary for starting the rooting process (Acosta et al., 2009) and often this can be facilitated by exogenous application of auxins in recalcitrant species. Meanwhile, recent studies indicate that basipetal auxin transport and auxin accumulation in the rooting zone may be negatively regulated by strigolactones (Rasmussen et al., 2012). This phytohormone class could act reducing auxin levels in the pericycle, decreasing root initiation. This could be a direct effect or via regulation of the amount of local auxin levels, presumably involving impairment of the rooting zone (Rasmussen et al., 2012). Thus, although auxin is the main hormone involved in AR, it clearly does not act alone, since crosstalk between several phytohormones is necessary for the success of this process.

## AUXIN RECEPTORS AND ACTION MECHANISMS

Even though auxin is known to play a central role in AR, the specific mechanisms of auxin action in this process are far from being understood. However, considering plant development in general, in the past decade a vast amount of data was reported regarding auxin perception (Mockaitis and Estelle, 2008). At the cellular level, auxin induces various rapid changes in cell physiology, such as membrane depolarization, apoplast acidification, cell wall loosening, activation of plasma membrane ATPases, and control of gene expression (Scherer, 2011). Although many of the signaling pathways leading to the responses mediated by auxin are still to be elucidated, significant knowledge on nuclear receptors for auxin is available. In the recent literature two different proteins are accepted as true auxin receptors, ABP1 and TIR1/AFB (auxin signaling F-box) proteins. The TIR1/AFB-family of F-Box protein members were the first authentic auxin receptors to be discovered (Dharmasiri et al., 2005a; Kepinski and Leyser, 2005). These proteins form nuclear regulatory complexes called SCF-E3-ubiquitin ligases and are responsible for the targeted degradation of a family of transcriptional repressors called AuxIAA proteins (Gray et al., 2001).

**FIGURE 3:**
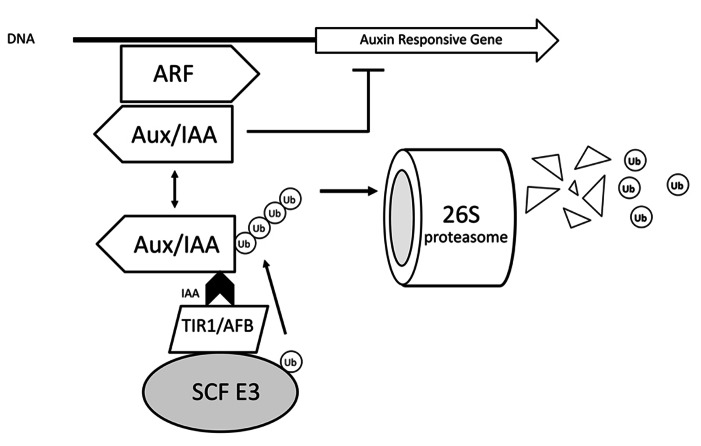
**Auxin action mechanism based on TIR1**. Upon binding of auxin to the F-Box (TIR1/AFB) subunit of the SCF TIR1/AFB complexes, their affinity toward the domain II of AuxIAA proteins is greatly enhanced with auxin acting as a “molecular glue” bringing the two proteins together; this binding triggers the ubiquitination of the AuxIAA by the SCF complex leading to its destruction by the 26S proteasome. The degradation of the transcriptional repressor releases the transcriptional activity of ARFs and auxin-responsive genes are expressed. E3 – ubiquitin protein ligase.

AuxIAA proteins are transcriptional repressors that act via dimerization with auxin-responsive transcription factors called ARFs (auxin-responsive factors). Upon binding of auxin to the F-Box (TIR1/AFB) subunit of the SCF TIR1/AFB complexes, their affinity toward the domain II of AuxIAA proteins is greatly enhanced with auxin acting as a “molecular glue” bringing the two proteins together; this binding triggers the ubiquitination of the AuxIAA by the SCF complex leading to its destruction by the 26S proteasome (Tan et al., 2007; Chapman and Estelle, 2009; Maraschin et al., 2009). The degradation of the transcriptional repressor releases the transcriptional activity of ARFs and auxin-responsive genes are expressed (**Figure 3**). The control of AR in intact seedlings of *Arabidopsis* by auxin, for example, involves activation of transcription factors *ARF6* and *ARF8* (Gutierrez et al., 2009). The TIR1/AFB family of auxin receptors is composed of 6 distinct members in *Arabidopsis* (namely TIR1, AFB1, AFB2, AFB3, AFB4, and AFB5), all of which are able to bind auxins specifically and show auxin-enhanced binding to AuxIAA proteins (Mockaitis and Estelle, 2008). Although much of the phenotypes of TIR1/AFB mutants indicate a large degree of redundancy, some specific features have already been identified. For example, TIR1 and AFB2 display a higher affinity for AuxIAA proteins compared to other members.

On the basis of the phenotype of single mutants, TIR1 appears to make the largest contribution followed by AFB2. Both AFB1 and AFB3 contribute to auxin response, but this contribution is only apparent in higher order mutant combinations. The *afb4* and *afb5* mutants are more resistant than *tir1* to picolinate auxins such as picloram, suggesting alternative substrate specificity (Parry et al., 2009). All of the defects observed in *afb4-2* mutant seedlings can be simulated in wild-type seedlings by treatment with auxin, indicating that AFB4 acts as a negative regulator of auxin-dependent processes. The *afb4-2* mutants have shorter roots and display a higher lateral roots/primary root length ratio than wild-type seedlings, suggesting that AFB4 has a role in anchor or adventitious root production (Greenham et al., 2011).

The expression patterns of the TIR/AFB genes are highly overlapped and not auxin-responsive, with the most significant regulation so far described being due to post-translational repression of TIR1, AFB2, and AFB3 by miR393 upon pathogen attack (Navarro et al., 2006). The structural specificity of auxin binding to TIR1 has been investigated to atomic level via X-ray crystallography. The details of this interaction provided valuable information to understand the mechanism of binding and structural details for active auxins (Tan et al., 2007). Recently, intensive efforts have been successful in designing TIR1-specific auxin antagonists, such as BH-IAA (tert-butoxycarbonylaminohexyl-IAA) and auxinole (Hayashi et al., 2008, 2012). These molecules specifically interact with the auxin-binding pocket on the TIR1 protein, blocking the access to the domain II of AuxIAAs. By testing the effects of blocking TIR1/AFB responses one is able to determine the contribution of TIR1/AFB-dependent transcriptional responses on whole plant phenotypes such as adventitious root formation. Although such inhibitors were designed based on the *Arabidopsis* TIR1 protein, the conservation of the TIR/AFB-AuxIAA mechanism goes all the way to mosses such as *Physcomitrella* sp., broadening the application of chemical tools to investigate physiological events in many unrelated plant species. A scheme on the TIR1 model of auxin action is shown in **Figure 3**.

Auxin-Binding Protein 1 was the first auxin-binding protein discovered, about 40 years ago (Hertel et al., 1972). ABP1 binding to auxin is highly specific and pH-dependent. Null *abp1* mutants are embryo-lethal and the functions of ABP1 on auxin signaling remained obscure since its discovery (Tromas et al., 2010). With the analysis of multiple TIR1/AFB mutants it became clear that nuclear perception of auxin and the degradation of AuxIAAs cannot account for all auxin-dependent cellular responses (Dharmasiri et al., 2005b). It is believed that plasma membrane localized ABP1 acts as an extracellular auxin receptor inducing rapid responses on the membrane and cytosol (Shi and Yang, 2011). The mechanism through which ABP1 is able to transduce the auxin signal to other molecules is still unknown. ABP1 has emerged as the receptor responsible for fast, protein synthesis independent, membrane and cytosolic responses to extracellular auxin concentrations. Many early auxin-dependent responses are attributed to ABP1 signaling: a fast (few milliseconds) drop on plasma membrane polarization, K^+^ influxes (0.5 s), rise in cytosolic Ca^2+^ (30 s), phospholipase A activation (2 min), MAPK activation (5 min), among other rapid auxin-triggered responses (Tromas et al., 2010). Recently, it has been demonstrated that auxin binding to ABP1 is able to inhibit clathrin-dependent PIN protein endocytosis at the plasma membrane (Robert et al., 2010). It has been proposed that ABP1 would be the receptor to regulate auxin transport throughout the plant whereas the TIR1/AFB proteins would be the receptors responsible for intracellular auxin transcriptional responses.

**FIGURE 4:**
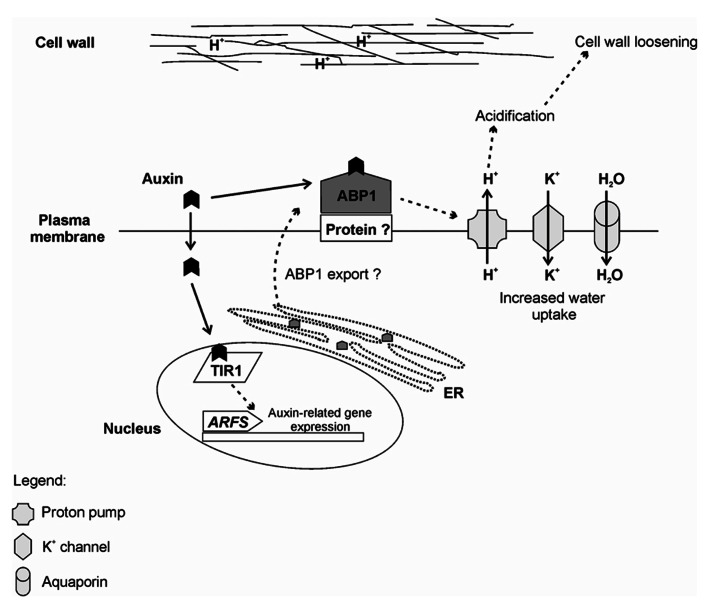
**Summary of the auxin perception by ABP1 and TIR1 receptors**. Rapid auxin responses are thought to be mediated by ABP1. Auxin is perceived by ABP1 at the outer surface of the Plasma membrane. In this case, ABP1 is anchored by an unknown membrane-associated protein (Protein?). In flowering plants, ABP1 is mainly located at the endoplasmic reticulum (ER) due to an ER retention motif (KDEL). Currently, it is not known how ABP1 is exported to the plasma membrane. Binding of auxin to ABP1 induces several events including activation of proton pumps, which culminates in acidification of the outer space and contributes to cell wall loosening. There is also activation of potassium inward channels, which increase the intracellular K^+^ and, consequently, lead to increased water uptake, allowing cell expansion. For slower responses, auxin is perceived by the F-box TIR1, which directs the auxin repressors Aux/IAAs for degradation and releases the auxin response factors (ARFS) to induce auxin-related gene expression.

The current scenario suggests that ABP1 and TIR1/AFB proteins are components of a two-receptor mechanism for auxin responses (Scherer, 2011) with ABP1 being an early sensor of apoplastic auxin concentrations regulating auxin transport and early, fast, transcriptional-independent, membrane and cytosolic responses, such as apoplast acidification and early elongation. TIR1/AFB would be the receptors responsible for the perception of nuclear and cytosolic auxin concentrations, involved in later, long term developmental responses, triggering transcriptional adaptive responses to the signal input generated by the ABP1-regulated auxin transport (Scherer, 2011). The relative participation of these auxin receptors in AR is currently unclear, but could putatively require sequential and conjunct activity in a rooting phase-dependent fashion. A putative model of ABP1 action and its interaction with TIR1 is shown in **Figure 4**.

## miRNA CIRCUITRY

Several miRNAs were reported as involved in root development modulation, reinforcing the growing awareness that miRNAs play pivotal roles in many biochemical or biophysical processes *in planta* (Meng et al., 2010). Gutierrez et al. (2009) established that microRNAs miR160 and miR167 were implicated in adventitious root formation through auxin signal further transduced by their downstream ARF targets (Meng et al., 2010). ARF6 and ARF8 targeted by miR167 were shown to be positive regulators of shoot-borne root emergence, whereas ARF17, a target of miR160, was a negative regulator (Gutierrez et al., 2009). ARF17 affects both miR167-dependent and independent regulation of ARF6 and ARF8. Conversely ARF6 represses ARF17 by activating miR160, whereas ARF8 directly represses ARF17. Finally, miR167 and miR160 appear to have opposite roles in controlling the expression of the auxin homeostatic enzyme GH3, which are required for fine-tuning adventitious root initiation in the *Arabidopsis thaliana* hypocotyl, acting by modulating JA homeostasis (Gutierrez et al., 2012). Thus miR160 targets reduce active auxin and AR, whereas miR167 targets act in opposite way (Rubio-Somoza and Weigel, 2011).

## ROOT GROWTH AND EMERGENCE THROUGH THE STEM

Adventitious root primordia, with apical meristem and differentiation of the basic root body, are formed and grow through the cortex toward the surface of the stem. Ethylene seems to be important to induce cell wall loosening and facilitate root passage through the stem tissues (Vidoz et al., 2010). Once newly formed roots reach the surface of the stem, a disruption of the epidermis and additional cell wall loosening take place, leading to root emergence. Afterward, the stem itself develops a periderm around the opening of each of the adventitious roots formed, important for protection against microorganism attack and drought (Hatzilazarou et al., 2006). The vascular reconnection between newly formed roots and the shoot is then fully established, allowing root nutrition, hydration, and growth (Hatzilazarou et al., 2006). In this process of vascularization and vascular connection, auxins and cytokinins are relevant for phloem and xylem tissue differentiation. In deepwater rice, a model has been proposed for phytohormonal interactions regulating root emergence. In this model, ethylene would promote epidermal programmed cell death, root emergence and elongation, and these processes would be co-stimulated by GAs and inhibited by ABA (Steffens et al., 2006).

## FINAL REMARKS AND PERSPECTIVES

In spite of a large volume of information on AR accumulated over the last few decades, a complete picture of this key developmental process is far from sight. Phytohormones are certainly at center stage in the conundrum of factors that influence AR. Not surprisingly, their actions involve significant degree of crosstalk, adding to the complexity of the process. In addition, a relevant participation of carbohydrate metabolism and mineral nutrition is evident, frequently modulating phytohormone-based controls. The wound response associated with the typical AR protocols add other players such as JA, H_2_O_2_, phenolics, and the action of enzymes on phytohormone content.

Faster advances of significant impact (both fundamental and practical) in the field of AR may depend on a number of strategies and scientific decisions for possible consideration by researchers. Although model species are a highly valuable tool for unveiling complex developmental processes, it is probably useful to somewhat diversify research objects, at least a couple of species for each general type of plant material (small herbaceous, monocots and dicots, horticulture/flower like crops, fruit crops, forest species, angiosperms, and gymnosperms) and within these seek for a few genotypes of easier or harder-to-root phenotype, in order to gain a better view of the process. A shift or at least a better balanced focus between research aiming at cuttings and at mother plant status and its implications on subsequent rooting may help achieve a more global understanding/predictable manipulation of AR. The recognition and identification of the main phases of AR should be taken into account in the various materials under investigation, for the process is quite dynamic and requisites and needs change along the process of re-establishing a root system.

From the experimental view point, solid associations must be established between structure and function, with a refinement of sampled cell types and tissues (cell/tissue-specific gene expression, proteomics, and metabolic profiling), always with a kinetic perspective of the successive phases. Another key association in the realm of methodologies is to maintain an open dialog between the basic and applied research with mutual benefits arising from exchanging operational strategies, investigation methods, and process modulation tools. Finally, a conjunct effort to establish clearer boundaries between lateral and adventitious root development and to seek an integrated look at these two processes within the various plant materials investigated may help clarify some of the contradictory data populating the rooting literature.

## Conflict of Interest Statement

The authors declare that the research was conducted in the absence of any commercial or financial relationships that could be construed as a potential conflict of interest.
